# The Nutrient Profiling of Swedish Food Products—A Study of the Alignment of the Multi-Level Criteria for the Choices and Nutri-Score Systems with the Nordic Keyhole Logo

**DOI:** 10.3390/nu17030421

**Published:** 2025-01-24

**Authors:** Wing Ki Chan, Stephanie Pitt, Herbert Smorenburg, Alicja Wolk, Lauren Lissner

**Affiliations:** 1 Department of Public Health and Community Medicine, Institute of Medicine, Sahlgrenska Academy University of Gothenburg, 40530 Gothenburg, Sweden; wing.ki.chan@gu.se; 2 Institute of Neuroscience and Physiology, Sahlgrenska Academy, University of Gothenburg, 40530 Gothenburg, Sweden; 3 Unit of Cardiovascular and Nutritional Epidemiology, Institute of Environmental Medicine, Karolinska Institutet, 17177 Stockholm, Sweden; stephanie.pitt@ki.se (S.P.); alicja.wolk@ki.se (A.W.); 4 Choices International Foundation, 7084 AV Breedenbroek, The Netherlands; herbert.smorenburg@choicesprogramme.org

**Keywords:** nutrient profiling system, NPS, multi-level criteria, choices criteria, Nutri-Score, front-of-package nutritional label, FOPNL, Keyhole, Sweden

## Abstract

Background: The European Commission has called for a harmonised front-of-package nutritional label (FOPNL) system in the European region. The Keyhole is a widely adopted positive FOPNL used in several Nordic countries. The Nutri-Score is a five-level graded FOPNL, more recently introduced in Western Europe. Both FOPNLs are supported by intrinsically different nutrient profiling systems (NPSs). A third NPS is the Choices NPS, which originally supported a positive FOPNL similar to the Keyhole and has been expanded into a five-level NPS. Objectives: The main objective of this study was to investigate the overall alignment between both the Choices and Nutri-Score multi-level NPSs and the Keyhole NPS. Furthermore, food group-based alignment was analysed to understand the main sources of misalignment. Methods: In this study, we extracted 1064 food/drink items from the food and beverage database of the Swedish Food Administration. All products were assessed according to all three NPSs, and alignment was assessed, using the Keyhole NPS as a recommendation-based reference. Two definitions of alignment were examined—healthier (more lenient, i.e., two highest grades) and healthiest (stricter, i.e., only the highest grade). Results: The overall alignment between the Choices and Keyhole NPSs was similar to that between the Nutri-Score and Keyhole NPSs (89% and 85% alignment under the healthiest definition, i.e., stricter). However, two food groups showed poor alignment between the Nutri-Score NPS and the Keyhole NPS (~60%). Under the healthier (more lenient) definition, the alignment for both the Choices and Nutri-Score systems with the Keyhole system was lower (86% and 79%). Conclusions: The alignments and misalignments between the Choices/Nutri-Score systems and the Keyhole system prompt important considerations for future developments of FOPNL systems for the Nordic countries. Extending the Keyhole NPS to include a multi-level criterion could potentially help consumers identify healthier choices, even for less nutritious foods.

## 1. Introduction

In 2020, as a part of the European Green Deal, the European Commission proposed an improvement in the food information that is available to customers. This included the introduction of a mandatory and harmonised front-of-pack nutrition label (FOPNL) in the European region to empower customers while encouraging product reformulation by the food industry [1]. FOPNLs are intended as a quick and simple means of conveying nutritional information about foods and beverages, with the downstream aim of reducing diet-related health conditions, including cardiometabolic diseases [2].

One of the global health issues that may be addressed by implementing effective FOPNL systems is the double burden of malnutrition, defined as “the coexistence of undernutrition alongside overweight and obesity… within individuals, households, populations, and across the life course” by the World Health Organisation (WHO) [3]. To address the underlying causes of the double burden of malnutrition and ensure nutrition security for all, this complex issue necessitates comprehensive and coordinated methods [4], with one of the possible means being the introduction of an appropriate and effective FOPNL supported by a Nutrient Profiling System (NPS) that is adaptive to the local diet and food habits.

In Europe, several FOPNLs have been introduced in recent decades. The Keyhole label, the longest-standing FOPNL in Europe, was launched as a voluntary initiative by the Swedish retailer ICA Gruppen in 1989 and was later adopted by the Swedish National Food Agency. This label evolved into a standardised Nordic Keyhole symbol promoting healthier food options, eventually being adopted by other Nordic countries, including Denmark, Norway, and Iceland, as well as by Lithuania and North Macedonia [5]. The Keyhole is a positive label displayed exclusively on healthier food options within pre-specified food groups/categories, excluding discretionary or ‘hedonistic’ foods and beverages such as sweets, snacks, and sugar-sweetened drinks. A cross-sectional study conducted six years after the Keyhole’s introduction in Sweden demonstrated an association between understanding the logo and higher consumption of Keyhole-labelled, low-fat food products [6]. A national survey in 2021 by the Swedish National Food Agency reported 97% recognition of the logo among study participants [7], indicating that the Keyhole logo is familiar to the majority of the Swedish population.

Another example of a positive FOPNL is the Choices logo. The Choices logo emerged in response to a call from the Dutch government for a standardised health logo and was first introduced in The Netherlands in 2006. It later expanded to Belgium, Poland, and the Czech Republic, as well as outside Europe, (e.g., Argentina and Nigeria) [8]. The use of the Choices logo was found to aid in food product classification to cater to the specific dietary requirements of different countries. It has also been found to enhance consumer knowledge, resulting in healthier customer choices [9]. The Choices logo also stimulated product reformulation by food companies to meet the standards for attaining a Choices logo [8]. The effects of the Choices logo on food product reformulation in The Netherlands were illustrated in another study conducted by Vyth et al. (2010) [9]. The implementation of the Choices logo was found to encourage product reformulation by food companies. Sodium and saturated fatty acid reductions, as well as dietary fibre increases, were reported across a variety of product categories, such as soups, snacks, processed meats, dairy products, and sandwiches. Reductions in added sugar and calorie content were also found in some food products [10]. Such findings were supported by a further, more recent study, which examined the effects of the Choices logo on food product reformulation in the Dutch market. It was found that products with a Choices logo had healthier nutritional values and the implementation of the Choices logo in The Netherlands had a positive effect on food reformulation over time [11]. Like the Keyhole label, the Choices logo was used on the healthiest products within each food group; however, it also included discretionary food categories. Due to concerns that displaying a positive logo on relatively healthier, but ‘hedonistic’ products could be misleading, the Choices logo was withdrawn from the Dutch market in 2016. Since then, the Choices International Foundation has not introduced a new FOPNL but has updated its NPS.

The Nutri-Score system, another prominent FOPNL, was developed by Santé Publique France, the French national public health agency, and launched in France in 2017. Since then, Belgium, Germany, Luxembourg, The Netherlands, Spain, and Switzerland have adopted the Nutri-Score [12]. In contrast to the positive logos used in the Keyhole and Choices systems, this graded label uses five colour-coded boxes, ranging from dark green to dark red, each labelled with letters (A to E) to denote a the overall nutritional quality of a product, from ‘A’ for the best nutritional quality to ‘E’ for the least favourable. The Nutri-Score logo is voluntary and can be applied to all product groups. Hercberg et al. (2022) described the features and overall aims of the Nutri-Score system in guiding healthier customer choices and motivating product reformulation by food manufacturers [13], which are consistent with the aforementioned proposal by the European Commission. Dréano-Trécant et al. (2020) investigated the application of the Nutri-Score in various European nations, including Finland, France, Norway, Poland, Portugal, Slovakia, Sweden, and Switzerland. The ability of the Nutri-Score to discriminate the nutritional content of foods and its conformity with national dietary recommendations were explored. The study revealed that the Nutri-Score system has a high discriminatory ability across all food classes, with similar trends across the eight countries and consistency with nutritional recommendations [14].

The WHO recently published a policy brief aimed at guiding policymakers in the development and implementation of FOPNL policies [15]. One of the WHO recommendations was that the FOPNL system should be aligned with national public health and nutrition policies (such as food-based dietary guidelines). Furthermore, FOPNL systems should be interpretive, offering consumers immediate insight into the healthfulness of a product. Interpretive systems include positive FOPNLs, such as the Keyhole and Choices logos, graded systems such as the Nutri-Score, and negative FOPNLs. The WHO does not endorse a specific system type; rather, it leaves the selection of an appropriate FOPNL to national policymakers. In order to develop and implement a harmonised FOPNL system across Europe, a key question to consider is whether the proposed FOPNL system is well aligned with national policies and guidelines. This requires a deeper understanding of the nutrition profiling system supporting the FOPNL.

### Nutrient Profiling Systems

Front-of-package nutrition labels (FOPNLs) visually represent a functional approach grounded in a nutrient profiling system (NPS). According to the WHO [2], nutrient profiling is ‘the science of classifying or ranking foods according to their nutritional composition for reasons related to preventing disease and promoting health’. While the NPSs for the Keyhole and Choices systems are similar, the Nutri-Score operates under a fundamentally different system.

Specifically, the Keyhole and Choices NPSs apply product group-specific threshold criteria, meaning that qualifying and disqualifying nutrients must all meet these standards for a product to be labelled. In contrast, the Nutri-Score system uses the same criteria across a few broad ranges of product groups and assigns positive and negative points based on the levels of qualifying and disqualifying nutrients. The sum of these points determines the Nutri-Score rating.

To accommodate positive, graded and negative FOPNLs with a product group-specific, threshold-based NPS, the Choices International Foundation expanded its NPS to a five-level system [16]. In this system, Level 1 represents the healthiest classification, while Level 5 indicates the least healthy. The Choices NPS differentiates between basic food groups—those typically included in dietary recommendations—and non-basic (discretionary or ‘hedonistic’) food groups. Similar to the Keyhole system, products within non-basic food groups would not be eligible for a positive FOPNL under this system. Unlike the Keyhole logo, which excludes all ‘hedonistic’ food products, the Choices criteria, as a multi-level NPS, are applicable for the assessment of all products including non-basic (‘hedonistic’) foods. According to the Choices criteria, non-basic food products are categorised into Levels C, D, or E (Levels 3, 4, or 5) [16,17], providing a detailed assessment of their relative healthiness or ‘unhealthiness’. This classification aids consumers in choosing basic foods and identifying ‘healthier’ options among non-basic foods, which can represent a substantial part of the diet.

The alignment between the NPSs mentioned above and national nutrition policies remains underexplored. Konings et al. [18] compared the Choices five-level criteria and the Nutri-Score NPS against the Dutch food-based dietary guidelines, finding that the Choices NPS aligned more closely with these national guidelines than the Nutri-Score system. Since then, the Nutri-Score NPS has undergone updates [19,20]. Previous research comparing the 2022 version of the Nutri-Score NPS with the Keyhole NPS, which is based on Nordic dietary guidelines, excluded beverages due to unavailable assessment criteria at that time [21].

The current study is distinguished from the aforementioned studies by introducing an additional, more restrictive (‘healthiest’), definition of alignment between the Keyhole and other NPSs, which may generate a new perspective for considering the most appropriate way of utilising an NPS to extend the Keyhole system, or any other positive FOPNL, into a graded system. This study incorporated the most updated version of the Nutri-Score (the Nutri-Score 2022 algorithm) in the comparison. This complements previous research in which the Keyhole system was compared with the Nutri-Score system, by allowing the evaluation of whether including beverage products influences the alignment between the Keyhole and Nutri-Score systems. 

The main objective of this study is to identify the most suitable NPS for extending a positive logo such as the Keyhole into a multi-level system. To achieve this, we consider the Keyhole system as a reference by comparing it with two different multi-level NPS frameworks for assessing Swedish food and beverage products. This study addresses key questions, such as how to define ‘healthier’ or ‘healthiest’, and whether it is more effective to have more specific or more generalised product groups in the context of a multi-level nutrition policy.

## 2. Materials and Methods

In this study, a total of 1,064 food and beverage products were extracted from the food database of the Swedish Food Agency (Livsmedelsverket) (version 2022-05-24) [22], consisting of 984 food products, which were previously assessed [21] and 80 additional beverage products, which were purposefully selected to include a wide range of diverse products. The ingredients and nutritional composition of each product were obtained from an ingredient database, which is available from by Livsmedelsverket upon request.

### 2.1. Assessment of Food Products

The assessment of food products was conducted based on the latest available version of all three assessment criteria. The Keyhole group allocation and assessment methodology has previously been described in detail [21]. Another element that was intentionally kept the same as in the previous publication is the Nutri-Score food group allocation of the products assessed [21]. For assessing the Nutri-Score of food and beverage products, a calculator tool released by Santé Publique France in September 2023 [12], which incorporated a revised algorithm specifically designed for evaluating beverage products, was used for assessing the newly added beverage products. A calculator tool specifically developed for Nutri-Score assessment in this study was also adopted for evaluating food products.

Lastly, the nutritional content information of food products was entered into an Excel calculator tool, developed by the Choices International Foundation, to assign the food products to the relevant Choices level. The determination of food products’ Choices level was performed by comparing the products’ nutritional content to four group-specific threshold values T1–T4, classifying the products in one of the five levels L1–L5 [16]. In the Choices NPS, industrially produced Trans Fatty Acids (iTFA) are a disqualifying nutrient for some product groups. Trans Fatty Acids (TFAs) occur naturally in animal source foods, but its labelling is not mandatory in Sweden [23]. As iTFA information is not available, it was excluded from the calculation and an assumption of 0 g of iTFA per 100 g of product content was made for all of the products for this study. The values for each threshold are stated in terms of per 100 g product content, except for the energy content of products in the Choices groups for sweet snacks, savoury snacks, main meals, and sandwiches and rolls, which are assessed based on both the energy content per 100 g of product content and per portion. As portion sizes were not available, the portion sizes of items in such food groups were not used. Instead, the Choices level assessment was conducted based on energy content per 100 g only. Further information regarding product classification according to the Keyhole, Nutri-Score and Choices criteria can be found in the Supplementary Material (Table S1).

### 2.2. Alignment Between Each of the Choices and Nutri-Score Criteria and the Keyhole Criteria

To assess how the alignment or misalignment between the Keyhole and Nutri-Score/Choices systems might differ under different circumstances, two definitions, namely the ‘healthier’ (i.e., more lenient) and ‘healthiest’ (i.e., stricter) definitions, were adopted for such assessment (Table 1). Using the Keyhole system as a reference point, the percentage of alignment and misalignment within each food group and overall was determined for both the Nutri-Score and Choices systems, under each definition of alignment.

### 2.3. ‘Healthier’ (i.e., More Lenient) Definition

In terms of alignment between the Keyhole and the Choices criteria, an alignment is said to be reached if (1) a product is assigned to either Choices Level 1 or Level 2 and is eligible for a Keyhole logo, or (2) a product is assigned to Choices Level 3, 4, or 5 and is ineligible for a Keyhole logo. Similarly, for the Keyhole and Nutri-Score, an alignment is reached if (1) a product is assigned to Nutri-Score letter A or B and is eligible for a Keyhole logo, or (2) a product is assigned to Nutri-Score level C, D, or E and is ineligible for a Keyhole logo.

### 2.4. ‘Healthiest’ (i.e., Stricter) Definition

For the assessment of alignment between the Keyhole and the Choices criteria under the ‘healthiest’ definition, an alignment is defined as (1) a product is assigned to Choices Level 1 and is eligible for a Keyhole logo, or (2) a product is assigned to Choices Level 2, 3, 4, or 5 and is ineligible for a Keyhole logo. In terms of the Keyhole and Nutri-Score systems, an alignment is considered if (1) a product is assigned to Nutri-Score letter A and is eligible for a Keyhole logo, or (2) a product is assigned to Nutri-Score letter B, C, D or E and is ineligible for a Keyhole logo.

To determine the level of alignment between the Keyhole system and an NPS in comparison (i.e., Choices or Nutri-Score systems), the formula of ‘(a + d)/n’, with ‘a’ and ‘d’ representing the criteria in which a case of alignment is reached and ‘n’ being the number of products in a particular group, was used. Conversely, to calculate the level of misalignment between the Keyhole and an NPS, we utilised the formula of ‘(b + c)/n’, with ‘b’ and ‘c’ being the criteria of a case in which a misalignment is found between the Keyhole system and an NPS. The details and definitions of each letter are summarised in Table 1. The overall alignment level between the Keyhole system and an NPS in comparison for each assessment approach was then calculated by dividing the number of products demonstrating an alignment by the total number of products in the database. All alignment levels are expressed as percentages.

## 3. Results

### 3.1. Overall Assessment of Product Healthfulness

**Figure 1:**
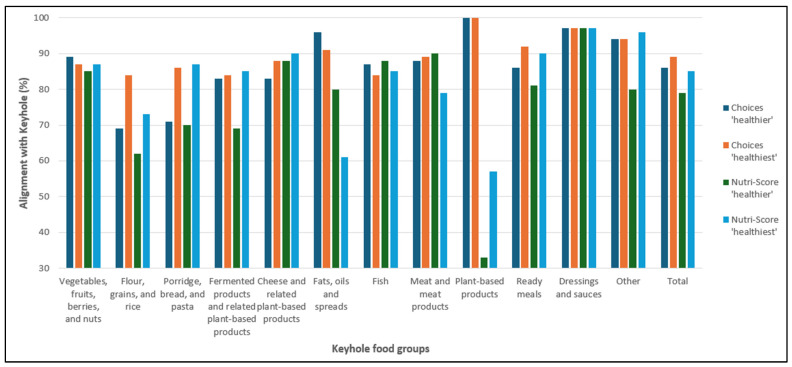
The level of alignment between the Keyhole system and each of the Choices and Nutri-Score definitions of alignment (‘healthier’ and ‘healthiest’) for each Keyhole food group and all food groups together.

Of the N = 1064 products assessed, 34% (n = 360) were determined as Keyhole eligible. Using the ‘healthier’ (i.e., more lenient) approach, 38% (n = 403) of the products were classified as Choices Level 1 or 2 and 47% (n = 499) were classified as Nutri-Score A or B. Considering the ‘healthiest’ (i.e., stricter) approach, 30% (n = 321) of the products were classified as Choices Level 1 and 34% (n = 359) were determined as Nutri-Score A (Table 2). In Figure 1, the level of alignment between the Keyhole system and each of the Choices and Nutri-Score definitions of alignment (‘healthier’ and ‘healthiest’) for each Keyhole food group and all food groups together is illustrated. The detailed results of the product assessment with the Keyhole (Table S2), Choices (Table S2), and Nutri-Score criteria (Tables S3 and S4) can be found documented in the Supplementary Materials.

### 3.2. Overall Alignment Between the Keyhole and Choices Criteria

Using the Choices ‘healthier’ (Choices Level 1 or 2) definition, 29% (n = 309) of the products were ‘healthier’ and were eligible for the Keyhole logo, and 57% (n = 610) were not ‘healthier’ and were Keyhole ineligible, resulting in an alignment of (309 + 610)/1064 = 86% between the Keyhole and Choices systems, for the ‘healthier’ classification. Among the 14% (n = 145) of products that demonstrated a misalignment, 9% (n = 94) were classified as ‘healthier’, but were ineligible for a Keyhole logo, while 5% (n = 51) were classified as not ‘healthier’, but were eligible for the Keyhole logo (Table 2). In terms of the products within each food group, 10 out of 12 groups had an alignment percentage of over 80%. The two groups with the highest levels of alignment were ‘Plant-based products’ (100%) and ‘Dressings and sauces’ (97%). The lowest levels of alignment were found in ‘Flour, grains and rice’ (69%) and ‘Porridge, bread and pasta’ (71%) (Figure 1 and Supplementary Material, Table S5).

Using the Choices ‘healthiest’ (Choices Level 1) definition, 26% (n = 281) were classified as ‘healthiest’ and were eligible for the Keyhole logo, whereas 62% (n = 664) were not ‘healthiest’ and ineligible for the Keyhole logo, resulting in an overall alignment of (662 + 281)/1064 = 89% between the Keyhole and Choices systems, for the ‘healthiest’ classification. Additionally, 11% (n = 119) of the products showed a misalignment: 4% (n = 40) were classified as ‘healthiest’ but were ineligible for a Keyhole logo, while 7% (n = 79) were classified as not ‘healthiest’ but were eligible for a Keyhole logo (Table 2). All food groups showed an alignment of at least 80% (Figure 1 and Supplementary Material, Table S6).

### 3.3. Alignment Between the Nutri-Score and Keyhole Systems

Using the Nutri-Score ‘healthier’ (Nutri-Score A or B) definition, 30% (n = 320) of the products were classified as ‘healthier’ and were eligible for a Keyhole logo and 49% (n = 525) were not ‘healthier’ and were ineligible for a Keyhole logo. This results in an overall alignment of (320 + 525)/1064 = 79% between the Keyhole and the Nutri-Score ‘healthier’ definitions. For the 21% (n = 219) of products that demonstrated a misalignment, 17% (n = 179) were assigned to the ‘healthier’ classification but were Keyhole ineligible, whilst the remaining 4% (n = 40) were classified as not ‘healthier’ but were Keyhole eligible (Table 2). The groups with the highest level of alignment between the two criteria were ‘Dressing and sauces’ (97%) and ‘Meat and meat products’ (90%). The groups with the lowest level of alignment were ‘Flour, grains, and rice’ (62%) and ‘Plant-based products’ (33%) (Figure 1 and Supplementary Material, Table S7).

Using the Nutri-Score ‘healthiest’ (Nutri-Score A) definition, there was an overall alignment of (279 + 624)/1064 = 85% between the Keyhole and the Nutri-Score ‘healthiest’ definitions, with 26% (n = 279) products having a Nutri-Score A and being eligible for the Keyhole logo, whilst 59% (n = 624) were classified as not ‘healthiest’ and were ineligible for the Keyhole logo. For the 15% (n = 161) of the products that demonstrated a misalignment, half (n = 80) were due to having Nutri-Score A but being Keyhole ineligible, whilst the other half (n = 81) had a Nutri-Score of B, C, D, or E, but were Keyhole eligible (Table 2). Regarding the food groups, eight groups showed an alignment level between the Keyhole and the Nutri-Score of above 80%. The two groups with the highest level of alignment were ‘Dressing and sauces’ (97%) and ‘Other’ (96%). The groups that demonstrated the lowest level of alignment between the Keyhole and the Nutri-Score systems were ‘Fats, oils and spreads’ (61%) and ‘Plant-based products’ (57%) (Figure 1 and Supplementary Material, Table S8).

### 3.4. Overall Alignment Between Each of the Choices and Nutri-Score Assessment Definitions and the Keyhole

Overall, the Keyhole has a slightly closer alignment with the Choices criteria than the Nutri-Score criteria. The highest level of alignment was found between the Keyhole and the Choices ‘healthiest’ level (89%), whereas the lowest level of alignment was found with the Nutri-Score ‘healthier’ approach (79%). There was a similar degree of alignment in the determination of Keyhole-eligible (more nutritious) products. The main difference between the Choices and Nutri-Score systems is in the assessment of Keyhole-ineligible (less nutritious) products (Table 2).

## 4. Discussion

### 4.1. Alignment of the Choices and Nutri-Score Criteria with the Keyhole System

The overall alignments of the Choices and Nutri-Score nutrient profiling systems (NPSs) with the Keyhole NPS were 80% or higher, representing a satisfactory result. The observed high level of alignment with both the Choices and Nutri-Score definitions of the ‘healthiest’ products suggests that the Keyhole NPS is relatively restrictive. However, of the three NPSs assessed, the Choices ‘healthiest’ classification remains the most restrictive.

The strong alignment between the Choices and Keyhole NPSs is not surprising, as both are based on product group-specific threshold criteria and have shared a collaborative history. Under the ‘healthier’ (more lenient) definition of alignment, the main discrepancies between the Choices and the Keyhole NPSs were for the groups of ‘Flour, grains, and rice’ (69%) and ‘Porridge, bread, and pasta’ (71%). The discrepancy in both food groups is due to the more lenient fibre requirement of the Choices Level 2 criteria than for the Keyhole criteria. Under the ‘healthiest’ definition, all food groups showed an alignment of at least 80%.

More notable, however, is the high level of alignment between the Nutri-Score and the Keyhole NPSs, given their intrinsically different approaches to nutrient profiling. In the product groups ‘Fats, oils and spreads’, ‘Porridge, bread, and pasta’, ‘Fermented products and related plant-based products’, and especially ‘Plant-based products’, however, these different approaches lead to very different assessments of healthiness. Under the ‘healthier’ (more lenient) definition, the alignment in these product groups varied between 33–70%, which is inadequate. Under the ‘healthiest’ (stricter) definition, the alignment for these product groups increased to ≥57%, but the alignment in the ‘Fats, oils and spreads’ group decreased from 80% (under the ‘healthier’ definition) to 61%. The alignment for these food groups would need to improve if the Nutri-Score system would be considered as a basis for a graded (Keyhole) FOPNL.

A few key factors emerged as assessment discrepancies between the Nutri-Score and Keyhole NPSs. As the Nutri-Score algorithm classifies products into a few broadly defined food groups, including ‘Solid Foods, Oils, Nuts, and Seeds’ (including cream products) and ‘Beverages’, each with a different set of assessment criteria, it is challenging to tailor the scoring mechanism for a specific food group without affecting the assessment of products from other food groups. Another potential factor is related to the ‘compensatory’ scoring methodology used in the Nutri-Score criteria, in which unfavourable components can be counteracted by a score for favourable components and vice versa.

One example of the potential impact of the ‘compensatory’ scoring approach of Nutri-Score is ‘Soy Protein Nuggets Cold or Frozen’ from the ‘Plant-based products’ group, which has the lowest alignment between the Nutri-Score and Keyhole systems. This product was ineligible for the Keyhole logo, and was assigned a Choices Level of 4 and a Nutri-Score of A. Within the Keyhole system, this product falls under the ‘For other products in group 25 (Plant-based products)’ category. Its salt content of 2.5 g per 100 g exceeded the maximum threshold of 1 g per 100 g, resulting in the product being ineligible for the Keyhole label. However, under the Nutri-Score system, the product was classified in the ‘Solid Foods’ group. It received an ‘A point’ score of 7 (2 points for energy content and 5 points for salt content) for unfavourable components and a ‘C point’ score of 7 (4 points for protein content, 2 points for fibre content, and 1 point for fruit and vegetable content) for favourable components. The difference between A points and C scores resulted in a final Nutri-Score of 0, corresponding to Level A. The assessment discrepancies between the Keyhole and Nutri-Score systems stem from the ‘compensatory’ scoring approach of Nutri-Score, where ‘unfavourable components’ (salt and energy content) are offset by ‘favourable components’ (protein, fibre, and fruit and vegetable content). This compensation resulted in the highest Nutri-Score level (A, 0 points). However, this methodology poses questions regarding the validity of the Nutri-Score’s assessment, as the inclusion of ‘favourable’ components may mask the impact of ‘unfavourable’ ones, potentially resulting in a problematic depiction of a product’s overall healthiness.

Previous research on the alignment between the Choices and Nutri-Score NPSs with the Dutch Wheel of Five [18] found similar levels of alignment for the Choices ‘healthier’ classification, as in this study, but significant lower alignment for the Nutri-Score ‘healthier’ classification. No comparison was done using the ‘healthiest’ definition. Since then, the Nutri-Score NPS has been revised, so that it would better align with the Dutch Wheel of Five. Previously, the Nutri-Score and Keyhole NPSs were compared using the ‘healthier’ definition of alignment and beverages were excluded [21]. The alignment between these two NPSs without beverages was 81%, similar to the 79% that was found here. We are not aware of other comparative studies evaluating the compatibility of different NPSs with national guidelines.

### 4.2. Points to Consider for the Future Development of an FOPNL in Sweden

The Keyhole is a well-established and trusted positive FOPNL empowering consumers to choose the most nutritious packaged food products on the market in Sweden and many other countries [7]. However, as the criteria are relatively strict and restricted to basic food groups, it does not help consumers with making healthier choices amongst less nutritious products. Graded FOPNLs such as the Nutri-Score system may do so. A public consultation conducted by the European Commission between December 2021 and March 2022 found that a graded indicator was considered the most useful for altering food purchasing behaviour and motivating food product reformulation by the industry [24]. On the other hand, the scientific evidence that the Nutri-Score FOPNL results in healthier food choices is debated [25,26,27,28,29]. The utilisation of a graded NPS, such as the Choices and Nutri-Score criteria, to inform the expansion of the current positive Keyhole logo into a graded FOPNL system could allow customers to identify the better options among the less nutritious options and minimise the adverse health effects of such products. While the main purpose of an FOPNL system is to encourage the consumption of healthy products, it is also crucial to guide customers in distinguishing between the different ends of the scale from more to less nutritious products, which the Keyhole system presently cannot do. However, expansion of the Keyhole system to a graded logo could incentivise the food industry to improve the nutrient quality of foods that are not deemed essential for a healthy diet. Furthermore, such a graded NPS can be used for other nutrition policies, such as restrictions on advertising to children and fiscal policies [16].

Despite the Choices and Nutri-Score criteria both being multi-level systems, the Choices criteria have group-specific nutritional thresholds, which could allow a more specific assessment of food products, based on their classification. The fact that only basic foods can receive a Choices Level 1 or 2 [16] can also prevent food companies from exaggerating or overestimating the overall nutritional value of a highly processed, non-basic product by adding favourable nutritional components. By making comparisons between the respective alignments between both the Choices and Nutri-Score systems and the Keyhole NPS, we identified some possible reasons behind certain similarities and differences in their alignment levels. The findings of this study could potentially reveal how the Choices and Nutri-Score criteria might support future developments of the Keyhole system, in response to the European Commission’s call for a harmonised FOPNL system in the European region.

### 4.3. Study Strengths and Limitations

This study has several strengths, including using a large number of food products from an official database published by the Swedish governmental food agency, which enhances the validity of the findings. This study also utilises the most recently published Nutri-Score 2022 algorithm and criteria for assessing beverages, allowing for a more complete set of food products to be included and assessed. Additionally, by using two definitions of alignment, a more nuanced perspective can be taken, providing alternative strategies for a harmonised FOPNL system. However, there are several limitations to this study. Firstly, the number of food products varied across different food groups. Future studies could focus on expanding the representation of food groups with fewer products to ensure a comprehensive analysis. Another limitation of this study is that, as the Livsmedelsverket database does not include all necessary nutritional information for the Choices assessment of some products, the TFA criteria were ignored for all products and only the energy density, and not the portion size criteria, were used to assess products from the ‘sweet snacks’, ‘savoury snacks’, and ‘main meal’ Choices groups. This could have affected the Choices levels that was calculated and therefore influenced the Keyhole–Choices alignment that was assessed, which could be addressed by supplementing the current Livsmedelsverket database with the missing information.

## 5. Conclusions

In conclusion, this study primarily focused on the level of alignment between the Choices multi-level criteria and Nutri-Score 2022 algorithm relative to the Keyhole NPS, in assessing Swedish food products. Building upon previous research that compared the Keyhole system to multi-level NPSs, such as the Dutch Wheel of Five and the prior version of the Nutri-Score (excluding beverages), this study adopted the most up-to-date Nutri-Score 2022 algorithm, including beverages. We have also included a third NPS for comparison, namely, the Choices criteria, which is fundamentally different from the Nutri-Score NPS in terms of both product classification and assessment methodology, to assess the respective agreement of each NPS with the Keyhole system and therefore its appropriateness for potential utilisation as the basis of extending the Keyhole system into a multi-level assessment criterion. For the first time, this study introduced an additional and more restrictive definition (the ‘healthiest’ definition) for assessment alignments between the Keyhole system and each NPS, as well as the assessment criteria used in previous studies (which correspond to the ‘healthier’ definition used in this study).

With the newly introduced ‘healthiest’ definition, both the Choices and Nutri-Score NPSs had a higher level of alignment with the Keyhole system than that based on the ‘healthier’ definition as used in previous studies, which assessed the alignment of the Keyhole system with the Wheel of Five and the prior version of the Nutri-Score system (excluding beverages) when evaluating products in the Dutch and Swedish food database. The results show that the best overall alignments of both the Choices and Nutri-Score systems with the Keyhole system, 89% and 85%, respectively, are obtained under the ‘healthiest’ definition. Despite the overall satisfactory alignment levels of the Keyhole system with both the Choices and Nutri-Score criteria, the alignment was somewhat stronger with the Choices system. For two food groups, the alignment between the Nutri-Score and the Keyhole system was ~60%, whilst between the Choices and the Keyhole system, all food groups showed an alignment of at least 80%. These findings suggest the potential of both Choices and Nutri-Score systems as reference NPSs for developing FOPNLs.

By extending the Keyhole system into a multi-level NPS, customers may be better guided in making informed, healthier choices among not only the healthier products but also the less healthy ones. The introduction of a multi-level NPS may also stimulate product reformulation by the food industry, potentially resulting in the enhanced availability of more nutritious food and beverage products. The methodology and findings of this study may contribute to supporting the extension of the Keyhole system into a multi-level FOPNL, not only in Sweden but also in other regions where it is adopted. Furthermore, this approach can be applied to the extension of other FOPNLs into multi-level systems and may also provide valuable insights for the development of marketing regulations and fiscal policies.

## Figures and Tables

**Table 1:** The criteria for reaching an alignment or misalignment between each of the Choices and Nutri-Score criteria and the Keyhole criteria. Letters ‘a’ and ‘d’ represents the criteria in which a case of alignment is reached between the Keyhole system and the NPS in comparison (Choices or Nutri-Score). Letters ‘b’ and ‘c’ represents the criteria of a case in which a misalignment is found.

	Keyhole Criteria			
	‘Healthier’ (more lenient) definition	‘Healthiest’ (stricter) definition	Keyhole eligible	Keyhole ineligible
Nutri-Score	A or B	A	a. Align	b. Misalign
	C, D, or E	B, C, D, or E	c. Misalign	d. Align
Choices	Level 1 or 2	Level 1	a. Align	b. Misalign
	Level 3, 4, or 5	Level 2, 3, 4, or 5	c. Misalign	d. Align

**Table 2:** The respective levels of alignment of the Choices and Nutri-Score ‘healthier’ (more lenient) and ‘healthiest’ (stricter) with the Keyhole, presented in descending order of their overall alignment with the Keyhole system.

				Keyhole Eligible		Keyhole Ineligible		Alignment with the Keyhole		
		n	% of N	n	% of N	n	% of N	Overall	Keyhole Eligible	Keyhole Ineligible
Database	N = 1064	1064	100%	360	34%	704	66%			
Choices	Healthiest	321	30%	281	26%	40	4%	89%	78%	94%
	Not healthiest	743	70%	79	7%	664	62%			
	Healthier	403	38%	309	29%	94	9%	86%	86%	87%
	Not healthier	661	62%	51	5%	610	57%			
Nutri-Score	Healthiest	359	34%	279	26%	80	8%	85%	78%	89%
	Not healthiest	705	66%	81	8%	624	59%			
	Healthier	499	47%	320	30%	179	17%	79%	89%	75%
	Not healthier	565	53%	40	4%	525	49%			

## Data Availability

This study utilised a food and beverage database that was publicly available via the following link: Food database, Swedish Food Agency (Livsmedelsverket): https://soknaringsinnehall.livsmedelsverket.se/ (accessed on 3 December 2023), or it can be requested directly from the Swedish Food Agency. The information regarding the ingredients and nutrient compositions of products in such database is available upon reasonable request to the Swedish Food Agency. The detailed results of this study can be found in the Supplementary Material (Files ‘Supplementary Tables S1, S5–S8’ and ‘Supplementary Tables S2–S4’).

## Supplementary Materials

The following supporting information can be downloaded at: https://www.mdpi.com/article/10.3390/nu17030421/s1, ‘Supplementary Tables S1, S5–S8’ and ‘Supplementary Tables S2–S4’. Table S1: A comparison of the respective food product classifications by the Keyhole, Choices, and Nutri-Score criteria [12,21,30,31]; Table S2: Keyhole and Choices assessment of food and beverage products, Table S3: Nutri-Score assessment of food products; Table S4: Nutri-Score assessment of beverage products; Table S5: The alignment between the Keyhole and Choices systems under the ‘healthier’ (more lenient) definition of alignment; Table S6: The alignment between the Keyhole and Choices systems under the ‘healthiest’ (stricter) definition of alignment; Table S7: The alignment between the Keyhole and Nutri-Score systems under the ‘healthier’ (more lenient) definition of alignment; Table S8: The alignment between the Keyhole and Nutri-Score systems under the ‘healthiest’ (i.e., stricter) definition of alignment.
