# Supplementary material for: The Nutrient Profiling of Swedish Food Products—A Study of the Alignment of the Multi-Level Criteria for the Choices and Nutri-Score Systems with the Nordic Keyhole Logo
Source: Nutrients. 2025 Jan 24;17(3):421. doi: 10.3390/nu17030421 (PMC11819656; doi:10.3390/nu17030421)
Supplement: Supplementary file 1 [file nutrients-17-00421-s001.zip › Supplementary Tables S1, S5-S8.pdf]

Table S1: A comparison of the respective food product classification by the Keyhole and the Choices and Nutri-Score criteria

The Keyhole			The Choices criteria		The Nutri-Score
**Food Group (group names used in our previous publication)**	**Food Category**	**Sub-category**	**Food Category**	**Food Group**	**Food Group**
Fishery products and products derived from these<br><br>(Fish, shellfish, and derivative products)	Fishery products and live mussels		Meat, Fish, Poultry and Eggs	Fresh, Frozen or Processed Seafood	Solid Foods
	Products produced from a minimum of 50% processed fishery products	Caviar and other tinned fish products			
		Sliced cold cut products.			
		Smoked or marinated fish.			
		Products not covered by food groups include 'sliced cold cut', 'smoked or marinated fish' or 'caviar and other tinned fish products'			
Meat and meat products<br><br>(Meat and meat products)	Unprocessed meat			Unprocessed meat, poultry and eggs	
	Meat and products containing meat	Raw products made from whole or carved pieces of meat that are surface marinated or seasoned		Processed meat and meat products	
		Raw or ready-to-eat products where minced or ground meat is the main ingredient		Insects	
		Ready for consumption or smoked products			

Fat spread and oils etc.<br>(Fat, oils and spreads)	Fat spreads and blends		Oils, fats and fat containing spread	Oils, fats and fat containing spread	Oils, nuts, and seeds (including cream products)
	Cooking oils, liquid fat spread and liquid blends				
Cheese and equivalent vegetable products<br><br>(Cheese and related plant-based products)	Cheese		Dairy	Cheese (-products)	Solid Foods
	Products which are wholly or partially of vegetable origin intended to be used as alternatives to products in the 'Cheese' group				
	Fresh cheese and equivalent products				
Milk, fermented products and vegetable alternatives etc.<br>(Fermented products and related plant-based products)		Milk and equivalent fermented milk products intended as a drink (may not be flavoured)		Milk (-products)	Beverages
		Vegetable products intended for the same use as products in the food group above (may not be flavoured)			
		Fermented milk products not intended to be drunk (may not be flavoured)			Solid Foods
		Vegetable products intended for the same use as products in the food group above (may be flavoured)			
		Fermented milk products not intended for drinking (may be flavoured)			
		Vegetable products intended for the same use as products in the food group above (may be flavoured)			
		Products comprising a mixture of milk and cream, intended as an alternative to cream, or equivalent			Oils, nuts, and seeds (including cream products)

		fermented products (must not be flavoured)			
		Products which are wholly or partially of vegetable origin intended for the same use as the products in the food group above (must not be flavoured)			
		Products comprising a mixture of milk and cream, intended as an alternative to cream, or equivalent fermented products (may be flavoured)			
		Wholly or partially vegetable products intended for the same use as the products in the food group above (may be flavoured)			Solid Foods
Porridge, bread and pasta etc.<br><br>(Porridge, bread and pasta)	Porridge and porridge powder		Sources of complex carbohydrates		Solid Foods
		Soft bread and bread mixes		Bread	
		Rye bread, bread mixes and other products based on rye			
	Hard bread, crusts and flour mixes				
	Pasta (without filling)			Plain noodles and pasta	
				Flavoured noodles and pasta	
Flour, grains and rice etc.	Cereal flour, flakes, grains and crushed cereal			Grains	
	Rice				

(Flour, grain and rice)	Breakfast flakes and muesli			Breakfast cereals	
Vegetables, fruit, berries and nuts etc.<br><br>(Vegetables, fruits, berries and nuts)	Potatoes, root vegetables, legumes (except peanuts) and other vegetables, unprocessed spices			Plain tubers used as staple	
				Processed tuber used as staple	
			Fruits and vegetables	Processed fruit	Solid Foods
				Processed vegetables	
				Processed beans and legumes	
	Unprocessed fruits and berries			Fresh or fresh frozen fruits and vegetables	
	Unprocessed nuts and peanuts		Nuts and seeds	Processed and unprocessed nuts and seeds	Oils, nuts, and seeds (including cream products)
Vegetable products (Plant-based products)	Partly or totally vegetable products with the same range of uses as fish and meat products	Sliced sandwich cuts			Solid Foods
		For other products in the same food group			
Ready meals etc. (Ready meals)	Ready meals with vegetables, a protein-containing part, and a carbohydrate-containing part		Meals	Main meals	
	Pirogues, pizzas, spring rolls, other pies than dessert pies and similar products				
	Sandwiches, baguettes, wraps and similar products			Sandwich and rolls	
	Soups			Soups	
	Dressings of oil and vinegar			Meal Sauces	

Dressings and sauces	Sauces			Emulsified Sauces	
(Other) *				Dark Sauces#	
				Other sauces (water-based)#	
				Savoury snacks#	
				Sweet snacks#	
				Fruit and vegetable juices#	Beverages
				Non-dairy milk substitutes#	
				Beverages#	
				All other products#	Solid Foods

A comparative table illustrating the respective food product classification by the Keyhole, the Choices and the Nutri-Score criteria. The asterisk (*) marks the 'Other' group, which was created for all products unclassifiable by the Keyhole. Some of the Keyhole food groups and sub-group names have been simplified. The hash (#) symbol marks the non-basic Choices food groups [11, 20, 30, 31]

Table S5: The alignment between the Keyhole and Choices under the 'healthier' (more lenient) definition of alignment

Keyhole Group	Number of products	Number of products with alignment		Number of products with misalignment		Overall % of alignment
		Choices Level 1 or 2 and Keyhole eligible	Choices Level 3, 4 or 5 and Keyhole ineligible	Choices Level 1 or 2, but Keyhole ineligible	Choices Level 3, 4 or 5, but Keyhole eligible	
Vegetables, fruits, berries, and nuts	221	163	34	20	4	89
Flour, grains, and rice	74	21	30	22	1	69
Porridge, bread, and pasta	90	5	59	16	10	71
Fermented products and related plant-based products	81	7	60	8	6	83
Cheese and related plant-based products	48	5	35	6	2	83
Fats, oils and spreads	46	16	28	0	2	96
Fish	68	38	21	1	8	87
Meat and meat products	121	51	56	4	10	88
Plant-based products	30	0	30	0	0	100
Ready meals	104	3	86	9	6	86
Dressings and sauces	37	0	36	0	1	97
Other	144	0	135	8	1	94
Total	1064	919		145		86
**Overall alignment between the Keyhole and Choices under the 'healthier' definition of alignment: 86%**						

The 'healthier' (more lenient) definition of alignment is Keyhole eligible and Choices Level 1 or 2; Keyhole ineligible and Choices Level 3, 4, or 5.

Table S6: The alignment between the Keyhole and Choices under the 'healthiest' (stricter) definition of alignment

**Keyhole Group**	**Number of products, n**	**Number of products with alignment**		**Number of products with misalignment**		**Overall % of alignment**
		Choices Level 1 and Keyhole eligible	Choices Level 2, 3, 4 or 5 and Keyhole ineligible	Choices Level 1 but Keyhole ineligible	Choices Level 2, 3, 4 or 5 but Keyhole eligible	
Vegetables, fruits, berries, and nuts	221	153	39	15	14	87
Flour, grains, and rice	74	17	45	7	5	84
Porridge, bread, and pasta	90	2	75	0	13	86
Fermented products and related plant-based products	81	6	62	6	7	84
Cheese and related plant-based products	48	4	38	3	3	88
Fats, oils and spreads	46	14	28	0	4	91
Fish	68	35	22	0	11	84
Meat and meat products	121	48	60	0	13	89
Plant-based products	30	0	30	0	0	100
Ready meals	104	2	94	1	7	92
Dressings and sauces	37	0	36	0	1	97
Other	144	0	135	8	1	94
Total	1064	945		119		89

Overall alignment between the Keyhole and Choices under the 'healthiest' (stricter) definition of alignment: 89%

The 'healthiest' (stricter) definition of alignment is Keyhole eligible and Choices Level 1; Keyhole ineligible and Choices Level 2, 3, 4, or 5.

Table S7: The alignment between the Keyhole and Nutri-Score under the 'healthier' (more lenient) definition of alignment

**Keyhole Group**	**Number of Products**	**Number of products with alignment**		**Number of products with misalignment**		**Overall % of alignment**
		Keyhole Eligible and Nutri-Score A or B	Keyhole Ineligible and Nutri-Score C, D, E	Keyhole eligible, but Nutri-Score C, D, E	Keyhole ineligible, but Nutri-Score A or B	
Vegetables, fruits, berries, and nuts	221	166	21	1	33	85
Flour, grains, and rice	74	21	25	1	27	62
Porridge, bread, and pasta	90	15	48	0	27	70
Fermented products and related plant-based products	81	6	50	7	18	69
Cheese and related plant-based products	48	4	38	3	3	88
Fats, oils and spreads	46	9	28	9	0	80
Fish	68	38	22	8	0	88
Meat and meat products	121	51	58	10	2	90
Plant-based products	30	0	10	0	20	33
Ready meals	104	9	75	0	20	81
Dressings and sauces	37	1	35	0	1	97
Other	144	0	115	1	28	80
Total	1064	845		219		79
**Overall alignment between the Keyhole and Nutri-Score under the 'healthier' definition of alignment: 79%**						

The 'healthier' (more lenient) definition of alignment is Keyhole eligible and Nutri-Score A or B; Keyhole ineligible and Nutri-Score C, D, or E.

Table S8: The alignment between the Keyhole and Nutri-Score under the 'healthiest' (i.e. stricter) definition of alignment

**Keyhole Group**	**Number of Products**	**Number of products with alignment**		**Number of products with misalignment**		**Overall % of alignment**
		Keyhole Eligible and Nutri-Score A	Keyhole Ineligible and Nutri-Score B, C, D, E	Keyhole eligible and Nutri-Score B, C, D, E	Keyhole ineligible and Nutri-Score A	
Vegetables, fruits, berries, and nuts	221	162	30	5	24	87
Flour, grains, and rice	74	19	35	3	17	73
Porridge, bread, and pasta	90	10	68	5	7	87
Fermented products and related plant-based products	81	4	65	9	3	85
Cheese and related plant-based products	48	3	40	4	1	90
Fats, oils and spreads	46	0	28	18	0	61
Fish	68	36	22	10	0	85
Meat and meat products	121	38	58	23	2	79
Plant-based products	30	0	17	0	13	57
Ready meals	104	7	87	2	8	90
Dressings and sauces	37	0	36	1	0	97
Other	144	0	138	1	5	96
Total	1064	903		161		85
**Overall alignment between the Keyhole and Nutri-Score under the 'healthiest' definition of alignment: 85%**						

The 'healthiest' (stricter) definition of alignment is Keyhole eligible and Nutri-Score A; Keyhole ineligible and Nutri-Score B, C, D, or E.
